# 2,4-Dichloro-*N*-(2,3-dichloro­phen­yl)benzene­sulfonamide

**DOI:** 10.1107/S1600536811040980

**Published:** 2011-10-08

**Authors:** Vinola Z. Rodrigues, Sabine Foro, B. Thimme Gowda

**Affiliations:** a Department of Chemistry, Mangalore University, Mangalagangotri 574 199, Mangalore, India; b Institute of Materials Science, Darmstadt University of Technology, Petersenstrasse 23, D-64287 Darmstadt, Germany

## Abstract

In the title compound, C_12_H_7_Cl_4_NO_2_S, the conformation of the N—C bond in the C—SO_2_—NH—C segment is *gauche* with respect to the S=O bonds. Further, the N—H bond in the C—SO_2_—NH—C segment is *syn* with respect to the *ortho*-Cl atoms in the aniline and sulfonyl benzene rings. The C—SO_2_—NH—C torsion angle is −51.98 (18)°. The sulfonyl and aniline benzene rings are tilted by 67.7 (1)° relative to each other. An intra­molecular N—H⋯Cl hydrogen bond occurs.

## Related literature

For the preparation of the title compound, see: Savitha & Gowda (2006 ▶). For hydrogen-bonding modes of sulfonamides, see: Adsmond & Grant (2001 ▶). For studies on the effects of substituents on the structures and other aspects of *N*-(ar­yl)-amides, see: Bhat & Gowda (2000 ▶), on *N*-(ar­yl)-methane­sulfonamides, see: Gowda *et al.* (2007 ▶), on *N*-(ar­yl)-aryl­sulfon­amides, see: Gelbrich *et al.* (2007 ▶); Perlovich *et al.* (2006 ▶); Gowda *et al.* (2009 ▶); Shetty & Gowda (2005 ▶) and on *N*-(chloro)-aryl­sulfonamides, see: Gowda *et al.* (2003 ▶).
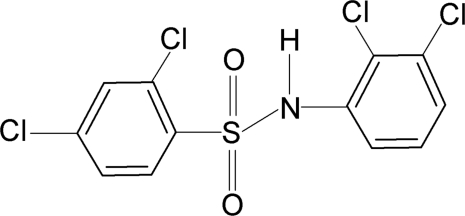


## Experimental

### 

#### Crystal data

C_12_H_7_Cl_4_NO_2_S
*M* *_r_* = 371.05
Monoclinic, 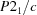

*a* = 9.0756 (6) Å
*b* = 9.7406 (7) Å
*c* = 16.432 (1) Å
β = 98.157 (6)°
*V* = 1437.92 (17) Å^3^
*Z* = 4
Mo *K*α radiation
μ = 0.97 mm^−1^
*T* = 293 K
0.48 × 0.48 × 0.40 mm

#### Data collection

Oxford Diffraction Xcalibur diffractometer with a Sapphire CCD detector
Absorption correction: multi-scan (*CrysAlis RED*; Oxford Diffraction, 2009 ▶) *T*_min_ = 0.654, *T*_max_ = 0.699
5584 measured reflections
2929 independent reflections
2377 reflections with *I* > 2σ(*I*)
*R*_int_ = 0.011

#### Refinement

*R*[*F* ^2^ > 2σ(*F* ^2^)] = 0.031
*wR*(*F* ^2^) = 0.085
*S* = 1.06
2929 reflections
184 parameters
1 restraint
H atoms treated by a mixture of independent and constrained refinement
Δρ_max_ = 0.35 e Å^−3^
Δρ_min_ = −0.33 e Å^−3^

### 

Data collection: *CrysAlis CCD* (Oxford Diffraction, 2009 ▶); cell refinement: *CrysAlis RED* (Oxford Diffraction, 2009 ▶); data reduction: *CrysAlis RED*; program(s) used to solve structure: *SHELXS97* (Sheldrick, 2008 ▶); program(s) used to refine structure: *SHELXL97* (Sheldrick, 2008 ▶); molecular graphics: *PLATON* (Spek, 2009 ▶); software used to prepare material for publication: *SHELXL97*.

## Supplementary Material

Crystal structure: contains datablock(s) I, global. DOI: 10.1107/S1600536811040980/ds2148sup1.cif

Structure factors: contains datablock(s) I. DOI: 10.1107/S1600536811040980/ds2148Isup2.hkl

Supplementary material file. DOI: 10.1107/S1600536811040980/ds2148Isup3.cml

Additional supplementary materials:  crystallographic information; 3D view; checkCIF report

## Figures and Tables

**Table 1:** Hydrogen-bond geometry (Å, °)

*D*—H⋯*A*	*D*—H	H⋯*A*	*D*⋯*A*	*D*—H⋯*A*
N1—H1*N*⋯Cl3	0.83 (2)	2.47 (2)	2.9526 (19)	118 (2)

**Fig. 1.**
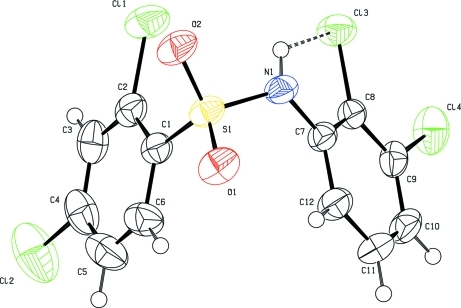
Molecular structure of the title compound, showing the atom labelling scheme and displacement ellipsoids are drawn at the 50% probability level.

**Fig. 2.**
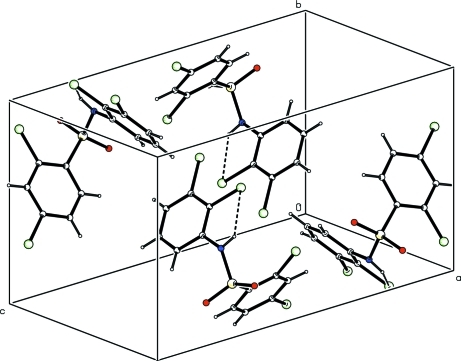
Molecular packing of (I) with hydrogen bonding shown as dashed lines.

## supplementary crystallographic information

### Comment

The sulfonamide moiety is the constituent of many biologically important compounds. The hydrogen bonding preferences of sulfonamides have been investigated (Adsmond & Grant, 2001). As part of our studies on the substituent effects on the structures and other aspects of *N*-(aryl)-amides (Bhat & Gowda, 2000), *N*-(aryl)-methanesulfonamides (Gowda *et al.*, 2007), *N*-(aryl)-arylsulfonamides (Gowda *et al.*, 2009; Shetty & Gowda, 2005) and *N*-(chloro)-arylsulfonamides (Gowda *et al.*, 2003), in the present work, the crystal structure of 2,4-Dichloro-*N*-(2,3-dichlorophenyl)benzenesulfonamide (I) has been determined (Fig. 1).

In (I), the conformations of the N—C bond in the C—SO_2_—NH—C segment is *gauche* to the S═O bonds. Further, the N—H bond in the C—SO_2_—NH—C segment is *syn* with respect to the *ortho*-Cl atoms in the anilino and sulfonyl benzene rings. The molecule is bent at the S atom with the C—SO_2_—NH—C torsion angle of -52.0 (2)°, compared to the value of -48.2 (2)° in 2,4-Dichloro-*N*-(3,4-dichlorophenyl)benzenesulfonamide (II) (Gowda *et al.*, 2009).

The sulfonyl and the aniline benzene rings are tilted relative to each other by 67.7 (1)°, compared to the value of 68.9 (1)° in (II).

The other bond parameters in (I) are similar to those observed in (II) and other aryl sulfonamides (Perlovich *et al.*, 2006; Gelbrich *et al.*, 2007).

The crystal structure exhibits N—H···Cl intramolecular hydrogen bonding. Part of the crystal structure is shown in Fig. 2.

### Experimental

The solution of 1,3-dichlorobenzene (10 ml) in chloroform (40 ml) was treated dropwise with chlorosulfonic acid (25 ml) at 0 ° C. After the initial evolution of hydrogen chloride subsided, the reaction mixture was brought to room temperature and poured into crushed ice in a beaker. The chloroform layer was separated, washed with cold water and allowed to evaporate slowly. The residual 2,4-dichlorobenzenesulfonylchloride was treated with 2,3-dichloroaniline in the stoichiometric ratio and boiled for ten minutes. The reaction mixture was then cooled to room temperature and added to ice cold water (100 ml). The resultant solid 2,4-dichloro-*N*-(2,3-dichlorophenyl)benzenesulfonamide was filtered under suction and washed thoroughly with cold water. It was then recrystallized to constant melting point from dilute ethanol. The purity of the compound was checked and characterized by recording its infrared and NMR spectra (Savitha & Gowda, 2006).

Prism like light pink single crystals used in X-ray diffraction studies were grown in ethanolic solution by slow evaporation at room temperature.

### Refinement

The H atoms of the NH groups were located in a difference map and later restrained to N—H = 0.86 (2) Å. The other H atoms were positioned with idealized geometry using a riding model with the aromatic C—H = 0.93Å and methyl C—H = 0.96 Å. All H atoms were refined with isotropic displacement parameters. The *U*_iso_(H) values were set at 1.2*U*_eq_(C-aromatic, N) and 1.5*U*_eq_(*C*-methyl).

### Crystal data

**Table:**

C_12_H_7_Cl_4_NO_2_S	*F*(000) = 744
*M**_r_* = 371.05	*D*_x_ = 1.714 Mg m^−^^3^
Monoclinic, *P*2_1_/*c*	Mo *K*α radiation, λ = 0.71073 Å
Hall symbol: -P 2ybc	Cell parameters from 2527 reflections
*a* = 9.0756 (6) Å	θ = 3.1–27.7°
*b* = 9.7406 (7) Å	µ = 0.97 mm^−^^1^
*c* = 16.432 (1) Å	*T* = 293 K
β = 98.157 (6)°	Prism, light pink
*V* = 1437.92 (17) Å^3^	0.48 × 0.48 × 0.40 mm
*Z* = 4	

### Data collection

**Table:**

Oxford Diffraction Xcalibur diffractometer with a Sapphire CCD detector	2929 independent reflections
Radiation source: fine-focus sealed tube	2377 reflections with *I* > 2σ(*I*)
graphite	*R*_int_ = 0.011
Rotation method data acquisition using ω scans	θ_max_ = 26.4°, θ_min_ = 3.1°
Absorption correction: multi-scan (*CrysAlis RED*; Oxford Diffraction, 2009)	*h* = −11→11
*T*_min_ = 0.654, *T*_max_ = 0.699	*k* = −12→6
5584 measured reflections	*l* = −20→7

### Refinement

**Table:**

Refinement on *F*^2^	Primary atom site location: structure-invariant direct methods
Least-squares matrix: full	Secondary atom site location: difference Fourier map
*R*[*F*^2^ > 2σ(*F*^2^)] = 0.031	Hydrogen site location: inferred from neighbouring sites
*wR*(*F*^2^) = 0.085	H atoms treated by a mixture of independent and constrained refinement
*S* = 1.06	*w* = 1/[σ^2^(*F*_o_^2^) + (0.0428*P*)^2^ + 0.4988*P*] where *P* = (*F*_o_^2^ + 2*F*_c_^2^)/3
2929 reflections	(Δ/σ)_max_ < 0.001
184 parameters	Δρ_max_ = 0.35 e Å^−^^3^
1 restraint	Δρ_min_ = −0.33 e Å^−^^3^

### Special details

**Table:**

Geometry. All e.s.d.'s (except the e.s.d. in the dihedral angle between two l.s. planes) are estimated using the full covariance matrix. The cell e.s.d.'s are taken into account individually in the estimation of e.s.d.'s in distances, angles and torsion angles; correlations between e.s.d.'s in cell parameters are only used when they are defined by crystal symmetry. An approximate (isotropic) treatment of cell e.s.d.'s is used for estimating e.s.d.'s involving l.s. planes.
Refinement. Refinement of *F*^2^ against ALL reflections. The weighted *R*-factor *wR* and goodness of fit *S* are based on *F*^2^, conventional *R*-factors *R* are based on *F*, with *F* set to zero for negative *F*^2^. The threshold expression of *F*^2^ > σ(*F*^2^) is used only for calculating *R*-factors(gt) *etc*. and is not relevant to the choice of reflections for refinement. *R*-factors based on *F*^2^ are statistically about twice as large as those based on *F*, and *R*- factors based on ALL data will be even larger.

### Fractional atomic coordinates and isotropic or equivalent isotropic displacement parameters (Å^2^)

**Table:**

	*x*	*y*	*z*	*U*_iso_*/*U*_eq_	
Cl1	1.01332 (7)	0.16349 (7)	0.03990 (4)	0.06229 (19)	
Cl2	0.68073 (12)	0.57632 (9)	−0.09497 (6)	0.0992 (3)	
Cl3	0.75598 (6)	−0.10291 (6)	0.09622 (4)	0.05174 (16)	
Cl4	0.41139 (7)	−0.13998 (7)	0.06337 (4)	0.06117 (19)	
S1	0.94908 (5)	0.25929 (6)	0.22226 (3)	0.03991 (14)	
O1	0.90401 (17)	0.33724 (18)	0.28772 (9)	0.0522 (4)	
O2	1.10217 (15)	0.22678 (18)	0.22388 (10)	0.0533 (4)	
N1	0.86340 (18)	0.11137 (19)	0.21735 (11)	0.0407 (4)	
H1N	0.907 (2)	0.052 (2)	0.1941 (13)	0.049*	
C1	0.8791 (2)	0.3472 (2)	0.13079 (12)	0.0381 (4)	
C2	0.9078 (2)	0.3066 (2)	0.05351 (13)	0.0420 (5)	
C3	0.8482 (3)	0.3780 (2)	−0.01612 (14)	0.0518 (6)	
H3	0.8692	0.3517	−0.0677	0.062*	
C4	0.7580 (3)	0.4880 (3)	−0.00799 (15)	0.0581 (6)	
C5	0.7266 (3)	0.5302 (3)	0.06718 (18)	0.0644 (7)	
H5	0.6646	0.6051	0.0714	0.077*	
C6	0.7884 (3)	0.4598 (2)	0.13660 (15)	0.0521 (6)	
H6	0.7688	0.4884	0.1880	0.063*	
C7	0.7057 (2)	0.0989 (2)	0.20112 (11)	0.0348 (4)	
C8	0.6430 (2)	−0.0002 (2)	0.14600 (11)	0.0357 (4)	
C9	0.4892 (2)	−0.0153 (2)	0.13160 (12)	0.0408 (5)	
C10	0.3983 (2)	0.0671 (3)	0.16999 (14)	0.0497 (6)	
H10	0.2955	0.0570	0.1594	0.060*	
C11	0.4611 (2)	0.1651 (3)	0.22439 (15)	0.0520 (6)	
H11	0.3998	0.2217	0.2506	0.062*	
C12	0.6134 (2)	0.1808 (2)	0.24075 (14)	0.0464 (5)	
H12	0.6542	0.2465	0.2784	0.056*	

### Atomic displacement parameters (Å^2^)

**Table:**

	*U*^11^	*U*^22^	*U*^33^	*U*^12^	*U*^13^	*U*^23^
Cl1	0.0608 (4)	0.0666 (4)	0.0633 (4)	0.0104 (3)	0.0221 (3)	−0.0190 (3)
Cl2	0.1211 (7)	0.0792 (6)	0.0854 (6)	−0.0130 (5)	−0.0259 (5)	0.0308 (4)
Cl3	0.0468 (3)	0.0479 (3)	0.0614 (4)	0.0047 (2)	0.0107 (2)	−0.0119 (3)
Cl4	0.0561 (3)	0.0688 (4)	0.0549 (3)	−0.0229 (3)	−0.0052 (3)	0.0012 (3)
S1	0.0291 (2)	0.0476 (3)	0.0424 (3)	−0.0015 (2)	0.00272 (19)	−0.0094 (2)
O1	0.0480 (9)	0.0646 (11)	0.0438 (8)	−0.0037 (8)	0.0059 (7)	−0.0187 (8)
O2	0.0271 (7)	0.0690 (11)	0.0625 (10)	−0.0014 (7)	0.0024 (6)	−0.0085 (8)
N1	0.0285 (8)	0.0427 (10)	0.0509 (10)	0.0034 (7)	0.0062 (7)	−0.0019 (8)
C1	0.0331 (10)	0.0361 (10)	0.0454 (11)	−0.0060 (8)	0.0062 (8)	−0.0092 (9)
C2	0.0360 (10)	0.0416 (11)	0.0493 (11)	−0.0095 (9)	0.0091 (9)	−0.0104 (10)
C3	0.0543 (13)	0.0543 (14)	0.0466 (13)	−0.0207 (11)	0.0062 (10)	−0.0030 (11)
C4	0.0650 (15)	0.0444 (13)	0.0601 (15)	−0.0169 (12)	−0.0079 (12)	0.0059 (12)
C5	0.0662 (16)	0.0376 (13)	0.0855 (19)	0.0047 (11)	−0.0028 (14)	−0.0026 (13)
C6	0.0571 (14)	0.0413 (12)	0.0579 (13)	0.0037 (10)	0.0076 (11)	−0.0120 (11)
C7	0.0292 (9)	0.0391 (11)	0.0364 (10)	0.0017 (8)	0.0056 (8)	0.0047 (8)
C8	0.0351 (10)	0.0364 (10)	0.0363 (10)	0.0029 (8)	0.0077 (8)	0.0078 (8)
C9	0.0356 (10)	0.0461 (12)	0.0395 (10)	−0.0064 (9)	0.0012 (8)	0.0114 (9)
C10	0.0267 (10)	0.0639 (15)	0.0591 (14)	0.0009 (10)	0.0081 (9)	0.0148 (12)
C11	0.0362 (11)	0.0587 (15)	0.0649 (15)	0.0070 (10)	0.0203 (10)	0.0001 (12)
C12	0.0405 (11)	0.0502 (13)	0.0505 (12)	0.0005 (10)	0.0135 (9)	−0.0053 (10)

### Geometric parameters (Å, °)

**Table:**

Cl1—C2	1.723 (2)	C3—H3	0.9300
Cl2—C4	1.729 (2)	C4—C5	1.370 (4)
Cl3—C8	1.720 (2)	C5—C6	1.380 (3)
Cl4—C9	1.734 (2)	C5—H5	0.9300
S1—O2	1.4216 (15)	C6—H6	0.9300
S1—O1	1.4232 (15)	C7—C12	1.384 (3)
S1—N1	1.6338 (18)	C7—C8	1.389 (3)
S1—C1	1.768 (2)	C8—C9	1.390 (3)
N1—C7	1.424 (2)	C9—C10	1.368 (3)
N1—H1N	0.825 (16)	C10—C11	1.375 (3)
C1—C6	1.383 (3)	C10—H10	0.9300
C1—C2	1.390 (3)	C11—C12	1.379 (3)
C2—C3	1.382 (3)	C11—H11	0.9300
C3—C4	1.366 (4)	C12—H12	0.9300
			
O2—S1—O1	119.32 (9)	C6—C5—H5	120.5
O2—S1—N1	105.16 (10)	C5—C6—C1	120.9 (2)
O1—S1—N1	108.79 (10)	C5—C6—H6	119.6
O2—S1—C1	110.86 (10)	C1—C6—H6	119.6
O1—S1—C1	106.09 (10)	C12—C7—C8	119.22 (18)
N1—S1—C1	105.90 (9)	C12—C7—N1	121.45 (18)
C7—N1—S1	122.92 (14)	C8—C7—N1	119.31 (17)
C7—N1—H1N	112.9 (17)	C7—C8—C9	119.53 (18)
S1—N1—H1N	112.7 (17)	C7—C8—Cl3	119.83 (14)
C6—C1—C2	118.7 (2)	C9—C8—Cl3	120.64 (16)
C6—C1—S1	118.10 (16)	C10—C9—C8	121.1 (2)
C2—C1—S1	123.21 (16)	C10—C9—Cl4	119.52 (16)
C3—C2—C1	120.7 (2)	C8—C9—Cl4	119.40 (17)
C3—C2—Cl1	117.35 (17)	C9—C10—C11	119.09 (19)
C1—C2—Cl1	121.91 (17)	C9—C10—H10	120.5
C4—C3—C2	118.9 (2)	C11—C10—H10	120.5
C4—C3—H3	120.5	C10—C11—C12	121.0 (2)
C2—C3—H3	120.5	C10—C11—H11	119.5
C3—C4—C5	121.9 (2)	C12—C11—H11	119.5
C3—C4—Cl2	119.2 (2)	C11—C12—C7	120.1 (2)
C5—C4—Cl2	119.0 (2)	C11—C12—H12	120.0
C4—C5—C6	119.0 (2)	C7—C12—H12	120.0
C4—C5—H5	120.5		
			
O2—S1—N1—C7	−169.42 (16)	C4—C5—C6—C1	−0.9 (4)
O1—S1—N1—C7	61.68 (19)	C2—C1—C6—C5	0.4 (3)
C1—S1—N1—C7	−51.98 (18)	S1—C1—C6—C5	−178.02 (19)
O2—S1—C1—C6	−136.46 (17)	S1—N1—C7—C12	−45.7 (3)
O1—S1—C1—C6	−5.5 (2)	S1—N1—C7—C8	136.06 (17)
N1—S1—C1—C6	109.99 (18)	C12—C7—C8—C9	0.0 (3)
O2—S1—C1—C2	45.2 (2)	N1—C7—C8—C9	178.29 (18)
O1—S1—C1—C2	176.12 (16)	C12—C7—C8—Cl3	−179.99 (16)
N1—S1—C1—C2	−68.37 (19)	N1—C7—C8—Cl3	−1.8 (3)
C6—C1—C2—C3	0.7 (3)	C7—C8—C9—C10	0.9 (3)
S1—C1—C2—C3	179.01 (16)	Cl3—C8—C9—C10	−179.07 (16)
C6—C1—C2—Cl1	−177.34 (17)	C7—C8—C9—Cl4	−179.51 (15)
S1—C1—C2—Cl1	1.0 (3)	Cl3—C8—C9—Cl4	0.5 (2)
C1—C2—C3—C4	−1.3 (3)	C8—C9—C10—C11	−0.8 (3)
Cl1—C2—C3—C4	176.82 (17)	Cl4—C9—C10—C11	179.58 (17)
C2—C3—C4—C5	0.8 (4)	C9—C10—C11—C12	−0.2 (3)
C2—C3—C4—Cl2	−179.01 (17)	C10—C11—C12—C7	1.1 (4)
C3—C4—C5—C6	0.2 (4)	C8—C7—C12—C11	−1.0 (3)
Cl2—C4—C5—C6	−179.9 (2)	N1—C7—C12—C11	−179.2 (2)

### Hydrogen-bond geometry (Å, °)

**Table:**

*D*—H···*A*	*D*—H	H···*A*	*D*···*A*	*D*—H···*A*
N1—H1N···Cl3	0.83 (2)	2.47 (2)	2.9526 (19)	118.(2)
